# Fractal biomarker of daily activity for women with early onset depression

**DOI:** 10.1136/bmjment-2024-301321

**Published:** 2025-06-10

**Authors:** Hui-Wen Yang, Mirjam Münch, Ma Cherrysse Ulsa, Arlen Gaba, Angelina Birchler-Pedross, Sylvia Frey, Vera Knoblauch, Peng Li, Sarah Laxhmi Chellappa, Christian Cajochen, Kun Hu

**Affiliations:** 1 Department of Biomedical Sciences and Engineering, Tzu Chi University, Hualien, Taiwan; 2 Division of Sleep and Circadian Disorders, Departments of Medicine and Neurology, Brigham and Women’s Hospital, Boston, Massachusetts, USA; 3 Centre for Chronobiology, Psychiatric Hospital of the University of Basel, Basel, Switzerland; 4 Department of Anesthesia, Critical Care and Pain Medicine, Massachusetts General Hospital, Charlestown, Massachusetts, USA; 5 Division of Sleep Medicine, Harvard Medical School, Boston, Massachusetts, USA; 6 School of Psychology, Faculty of Environmental and Life Sciences, University of Southampton, Southampton, UK

**Keywords:** Sleep, Depression & mood disorders, Depression

## Abstract

**Background:**

Depression is a major health issue in adolescence and young adulthood, emphasising the need for early risk identification. Patients with major depressive disorder (MDD) often show disturbed daily rest-activity patterns, but such changes are often confounded by medication intake, comorbidities and disease duration.

**Objective:**

In this exploratory analysis, we tested whether there are specific changes in daily rest activity (from wrist-worn actigraphy) in women at the onset of MDD without medication, as compared with age-matched controls.

**Methods:**

Participants from the MDD group (age 19–32, 24.73±5.13 (mean±SD), N=15) and control group (age 20–31, 24.89±3.82, N=9) completed ~7 day ambulatory actigraphy recordings, followed by a stringently controlled circadian laboratory protocol to assess endogenous circadian melatonin levels. We analysed the daily rhythm of mean activity levels and non-linear fractal dynamics in eight 3-hour time bin across the 24 hours, correlating these measures with depressive symptom severity and endogenous melatonin levels.

**Findings:**

Using approaches from non-linear fractal dynamics, we showed that, compared with healthy controls, women at MDD onset had a higher fractal activity correlation (FAC) during the last hours of sleep, indicating more ‘wake-like’ patterns (FAC within 0–3 hour before wake: 0.92±0.64 (SD) in MDD vs 0.77±0.18 in controls, p=0.02). The alteration was independent of mean activity level and wake duration but appeared to be associated with depressive symptom severity (p=0.08). Moreover, there was a trend association for altered FAC with endogenous melatonin levels in the MDD group (for onefold increase in melatonin level in the last 3 hours before wake, the FAC increased by 0.33±0.17 (SE), p=0.08).

**Conclusions:**

Pre-wake FAC is elevated in unmedicated women at MDD onset and may serve as a potential biomarker associated with symptom severity and circadian physiology.

**Clinical implications:**

These findings provide proof-of-concept evidence that unique fractal motor activity patterns may support early detection of MDD.

**WHAT IS ALREADY KNOWN ON THIS TOPIC**

- Patients with major depressive disorder (MDD) frequently experience disruptions in their daily rest-activity patterns. However, these disturbances are often masked by medication use, comorbidities and the duration of depressive episodes.

**WHAT THIS STUDY ADDS**

- Using novel non-linear fractal measures, we showed that the women with early and untreated MDD exhibit specific alterations in their daily rest-activity patterns (ie, higher fractal activity correlation), with more ‘wake-like’ activity patterns during the last 3 hours of sleep before waking up. Moreover, these changes tended to be associated with depression severity and the endogenous melatonin regulations.

**HOW THIS STUDY MIGHT AFFECT RESEARCH, PRACTICE OR POLICY**

- Adolescents and young adults, particularly women, are at increased risk for depression, which highlights the need for early detection. Using simple, non-invasive wrist-worn wearables combined with fractal analyses, this study provides unique insights into behavioural changes at early onset of MDD in young women. Remote and long-term physiological monitoring of activity patterns by wearable devices provides a cost-effective and scalable approach for early detection of depression.

## Background

 Adolescent and young adult depression are a major public health problem, with 4.4% of them experiencing depression, with a 2:1 prevalence in women compared with men.^1^ Compared with any other adult age group, depression is most prevalent among adolescents and young adults aged 18–25 years in the USA.^2^ More than 400 000 people die due to suicide every year in the USA, and it is one of the leading causes of death in adolescence and young adulthood.^3^ Thus, early identification is needed to help promote young mental healthcare.

Disturbances in sleep and circadian rhythms are bidirectionally intertwined with depression.^4^ Patients with depression often experience sleep and circadian rhythm disturbances (SCRD) due to a combination of psychological, neurobiological and behavioural factors.^5^ On the other hand, SCRD predict the onset of depression^6^ and are one of the earliest signs of depression relapse.^4^ Because SCRD have a causal role in depression,^5^ it may serve as modifiable risk factors for depression development and can be monitored with 24 hours at-home wrist-worn actimetry sensors (actigraphy). This wearable approach captures motor activity during wakefulness, estimates sleep outcomes at home and offers non-invasive, low-cost, long-term monitoring of depression. Using actigraphy, previous studies showed lower mean activity levels and altered 24-hour activity rhythms (eg, dampened amplitude and delayed phase) in individuals with major depressive disorder (MDD).^7 8^ However, such behavioural changes in MDD may be confounded by medication intake, disease duration and comorbidities, including anxiety disorders. In addition, mean activity level can be masked by external influences such as daily scheduled work, exercise and social activities.

Non-linear motor activity measures have been an emerging tool for studying psychiatric disorders^9^,^11^. Based on the interdisciplinary field, ‘fractal physiology’ has revealed that human motor activity in healthy young adults displays strong fractal fluctuations (ie, similar temporal correlation at different time scales).^12^ Fractal activity correlation is independent of mean activity level and environmental conditions.^12^ Compared with mean activity level-based measures of rhythmicity, fractal activity correlation appears to be more sensitive to intrinsic changes in ageing and dementia while being more resilient to external masking factors.^13^ Recent studies also showed fractal activity correlation in a 6-hour window during the day is excessively elevated in adult patients with MDD,^9^ indicating less ‘complex’ or too regular activity fluctuations; another study showed a smaller fractal activity correlation in patients with bipolar disorder, indicating more random activity fluctuations.^14^ Fractal activity correlation may help identify specific changes due to SCRD from 24-hour ambulatory activity recordings collected with simple, non-invasive wrist-worn wearables, facilitating the early detection of MDD in youth.

### Objective

We aimed to test whether at-home (wrist worn) daily rest-activity rhythms of fractal activity correlation and mean activity level could serve as potential indicators for the early detection of unmedicated patients at the onset of MDD. We hypothesise that fractal activity correlation in women with MDD differs from that in age-matched healthy women without MDD (control group) and is correlated with the severity of depressed symptomatology. As fractal activity correlations have yet to be tested in patients with MDD, we did not make any *a priori* assumptions about the direction of effects. We further explored whether temporal rest-activity fractal dynamics in MDD are linked to any changes in circadian regulation by examining the association of fractal activity correlation with endogenous circadian melatonin rhythms measured under a stringently controlled circadian protocol in the laboratory.

## Method

Our case–control study is comprised of (at least) 7-day ambulatory motor activity recordings and a 4-day laboratory study that included circadian constant routine protocols in young women with MDD and age-matched women without MDD.^15^ The associated methods are described briefly in this section and are described in detailed in online supplemental material. Other aspects of this study, which was designed to test separate, independent hypotheses, have been published previously.^15 16^

### Study design

We enrolled 15 unmedicated women who had first MDD episode within 2 years (age 19–32, 24.73±5.13 (mean±SD)), and 9 age-matched healthy women (age 20–31, 24.89±3.82) for the circadian laboratory study . Participants were first interviewed by the same clinical psychologist to ensure that they met the following criteria: they were experiencing an MDD episode and met DSM (Diagnostic and Statistical Manual of Mental Disorders)-IV diagnostic criteria^17^; had no atypical symptoms or other comorbid DSM-IV psychiatric disorders; had no long history of depression, with the current episode lasting <24 months; and had not received psychiatric treatment or used psychotropic drugs. We also exclude participants with severe sleep problems (Pittsburgh Sleep Quality Index, PSQI<8), classified as evening types,^18^ and had PSG-diagnosed sleep disorders during the adaptation night in the laboratory (detailed criteria listed in Frey *et al*^16^ and online supplemental material). During the clinical interview, the clinical psychologist also assessed depression scales including Hamilton-17 and the Montgomery-Åsberg Depression Scale (mean: 16.71±2.13 SD), and the Beck Depression Inventory (mean value 21.29±6.84 SD).

The first phase of the study involved (at least) 7 days of at-home rest-activity measurements, and participants were instructed to maintain a regular sleep–wake cycle with 8 hours of nighttime sleep for 7 days at self-selected target times of sleep within a range of ±30 min. Motor activity recordings were collected from these participants using wrist-worn activity wearables (Cambridge Neurotechnology, Cambridge, Cambridgeshire, UK). For the control group, seven of the nine participants had two recordings separated by 4 weeks.

Thereafter, participants underwent a 3.5-day laboratory experiment that included two baseline nights, a 40-hour circadian protocol, and a recovery night.

In the 40-hour circadian protocol, the participants with MDD were randomly allocated to two conditions: either sleep deprivation (SD, N=7) or multiple naps (NAP, N=8). In the SD condition, participants underwent 40-hour sleep deprivation in bed under constant conditions in dim light (<15 lx); in the NAP protocol, subjects experienced 10 cycles of 150 min scheduled wake (in bed, also in dim light) and 75 min scheduled sleep. In the control group, the participants were assigned to the SD protocol after one of the 7-day recording period. We used the data obtained from the 40-hour protocol for the endogenous melatonin level results. Accordingly, saliva samples were collected at a 30 min interval during the participant’s wake periods. Melatonin concentration was measured with a direct double-antibody radioimmunoassay with an analytical least detectable dose of 0.65 pm/mL (Bühlmann Laboratories, Schönenbuch, Switzerland).^16^

### Wrist-worn data analysis

**Figure 1:**
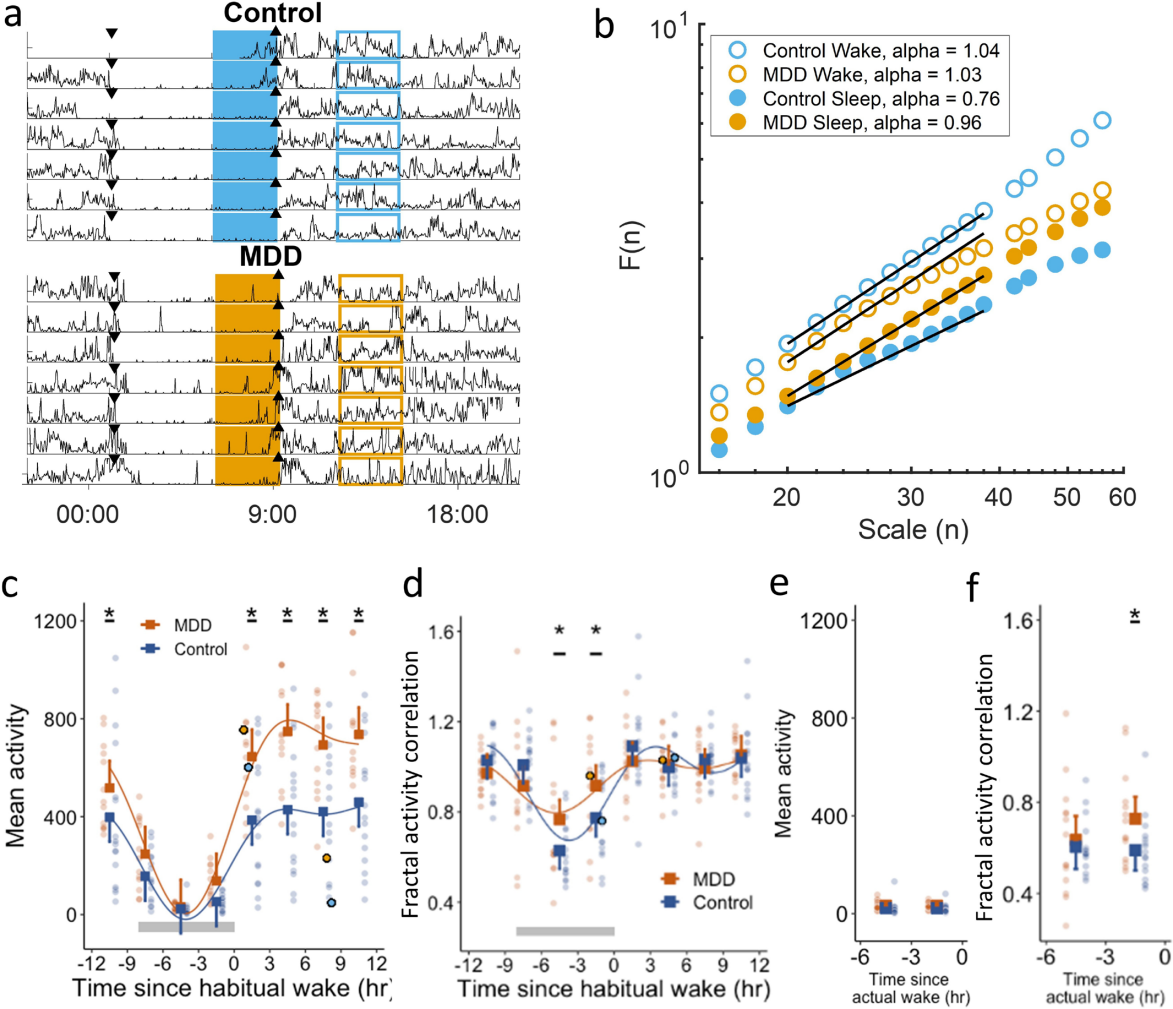
(**a**) Activity recordings of two representative participants from the control and MDD groups, respectively. Data were aligned by each participant’s habitual wake-up time and divided into 3-hour bin. Down-pointing/up-pointing triangles indicate habitual bedtime/wake-up. (**b**) Fractal analysis results obtained from the data within rectangle areas in (**a**); coloured shapes=during wakefulness; hollowed shapes=during sleep; orange=MDD; blue=control group. (**c–d**) Daily rhythms of mean activity levels (**c**) and fractal activity correlations (**d**). The rhythms (solid curves) were obtained from cosinor fitting with a 24 hours sinusoid and a 12 hours harmonic. Circles are the individual results obtained from the data within rectangle areas in (**a**). Grey horizontal bars are the average of habitual sleep periods. (**e–f**) Mean activity (**e**) and fractal activity correlation (**f**) within the last 6 hours before actigraphy-derived wake-up (actual wake-up). In (**c–f**), squares are the group means; error bars indicate 95% CIs; dots are the individual data. *p<0.05 for the group differences in the post-hoc comparison of the mixed model, including time, group and their interaction. MDD, major depressive disorder.

At home, 7-day actigraphy recordings were resampled into 2 min epochs, with activity counts in each epoch representing the integrated acceleration changes. In the first stage, we examined the daily rhythm of mean activity level and fractal activity correlations. An activity recording was first aligned by each participant’s habitual wake-up time, and the 24-hour sleep–wake cycle was divided into eight 3-hour non-overlapping bins (figure 1a). This bin size was selected to capture 24-hour rhythm in fractal activity patterns (ie, the rhythm would be less or not visible if the size was too large) while providing sufficient data points in each bin for reliable estimation of temporal correlations in the fluctuations (online supplemental figure S1 and S2).^19^ For each of the eight 3-hour bins across the 24-hour cycle, we pooled all 3-hour segments at different days (eg, total 21-hour data or 630 data points for a 7-day recording), calculated mean activity level and performed the detrended fractal analysis (DFA, figure 1b) to derive fractal activity correlation. The mean activity level in each 3-hour bin was calculated as the mean of all the activity counts within the bin. The fractal activity correlation is obtained by calculating alpha with DFA in the timescale range between 20 and 40 min (or 10–20 data points). This time scale is selected following the previous established guideline^19 20^ to ensure reliable estimation of temporal correlation in each 3-hour bin (detailed in online supplemental material).

The detailed procedure of DFA has been previously published.^21^ Subsequently, DFA analysis calculated the fluctuation amplitude function, F(n), of time scale (n)—square root of the residual sum of squares after second-order polynomial fitting of activity fluctuations within each bin of the same size equal to n. The slope, alpha, of this function on a log–log plot (ie, F(n) ~n^alpha^) indicates fractal activity correlation in activity fluctuations (figure 1b). The fractal activity correlation=0.5 indicates white noise, and larger alpha values indicate stronger temporal correlations.

To further reduce the influence from the variations in daily wake-up times, we repeated the analysis by aligning the data using the actual wake-up times determined by actigraphy for each day. We derived the mean activity level and fractal activity correlation within two 3-hour preactual wake-up bins: 3–6 hours and 0–3 hours before wake-up.

We also assessed typically derived ‘circadian’ measures of rest-activity rhythms, including cosinor analysis, interdaily stability, intradaily variability, the most active 10 hours (M10) and the least active 5 hours (L5), sleep/wake regularity index.^22^ We also determined the absolute deviation of actual wake-up time from habitual wake-up time (the average absolute value of the difference between daily actual bedtime from habitual wake time) and sleep quality indexes. These measures were assessed as exploratory analyses.

### Melatonin data analysis

For the melatonin records from the laboratory protocol, we first fitted the melatonin samples by a three-harmonic cosinor fitting, and the dim-light melatonin onset/offset (DLMO/DLMOff) is defined by the time at which the fitted curve rose above (DLMO) or fell below 25% (DLMOff) of the fitted peak-to-trough amplitude.^23^ We calculate the phase-angle between DLMO and actual bedtime (actual bedtime minuses the DLMO time), and DLMOff and actual wake-up time (actual wake-up time minuses the DLMOff time) for each day. In addition, we also calculated the average melatonin concentration during the last 3 hours of habitual sleep (MEL_last3hr). To reduce the individual variation and protocol differences (online supplemental figure S3), we normalised the melatonin concentrations of the last 3 hours by the average 24-hour melatonin concentrations, centred at the midpoint of the habitual bedtime and wakeup time.

### Statistical analysis

All the statistical analyses were performed using R (V.4.3.2). We performed two-way linear-mixed models with subjects as random effect to test the statistical significance of group effect on the daily rhythm of mean activity level and fractal activity correlation, separately. The group, time segment and their interaction are the fixed effects. If a significant interaction was observed (determined by p value <0.05), post-hoc analyses were performed to test the group difference within each 3-hour bin. The group differences in other conventional measures for sleep/circadian were also tested with a t-test or Mann-Whitney test if the test of normality failed at a value <0.05.

We further performed a test for correlation between the activity measure in the different time windows and depression level (as measured by MADR) to see if the actigraphy measure predicts severity of depression.

We investigated the group differences in the DLMO, DLMOff and MEL_last3hr by t-test or Mann-Whitney test. For the phase angle between DLMO and bedtime and DLMOff and wake-up time, we tested the group differences by linear mixed models, with group effect the fix factor and the participants as random factor (since the phase angles were derived from the actual bedtime and wake-up time for each day, one participant has multiple observations). The correlation between these melatonin measured and the MADR was tested by simple linear model. The potential correlation between melatonin measures and fractal activity correlation in the last 3-hour of sleep was also investigated.

## Findings

### Daily rhythm of mean activity level and fractal activity correlation

We examined mean activity level and fractal activity correlation in each of the eight 3-hour non-overlapping windows across the 24 hours (figure 1c,d). In both groups, mean activity level displayed a daily rhythm (p<0.0001) with higher levels during habitual wakefulness and lower levels during habitual sleep (figure 1c). Fractal activity correlation also showed a daily rhythm in both groups (p<0.0001), that is, weaker fractal activity correlation during habitual sleep, indicating or more random activity fluctuations, than during habitual wakefulness (figure 1d). Consistent rhythms of mean activity level and fractal activity correlation were also identified across actigraphy-derived sleep–wake cycles, that is, mean activity level was lower and fractal activity correlation was weaker during sleep (mean activity level=580.10±232.00 (SD) during wakefulness and 91.24±50.00 during sleep; fractal activity correlation=1.04±0.14 during wakefulness and 0.75±0.20 during sleep; both p<0.0001). As compared with healthy controls, women with MDD had higher mean activity level within the five 3-hour bins during habitual wakefulness (all p<0.001, figure 1c) but similar activity levels during habitual sleep (all p>0.05). In contrast, fractal activity correlation differed between groups within the last two 3-hour bins of the habitual sleep period (3–6 hour before wake: MDD: alpha=0.77±0.26, control: 0.63±0.13, p=0.02; 0–3 hours before wake: MDD: 0.92±0.64, control: 0.77±0.18, p=0.02). These results indicate that activity patterns in the MDD group before habitual wake-up more resembled those during wakefulness. When aligning the wake-up time identified from daily actigraphy, mean activity level during sleep remained similar between groups (figure 1e), while the group difference in the fractal activity correlation remained during the last 3 hours of sleep (ie, larger alpha in MDD, p=0.04) but became not significant in the bin of 3–6 hour before wake-up (p=0.26) (figure 1f).

### Group differences in other sleep/circadian-related measures

We tested whether there were group differences in typically assessed sleep/circadian-related measures, as exploratory analyses (table 1). Only two measures differed between groups: the absolute deviation between actigraphy-derived wake-up time and habitual wake-up time (MDD: 0.95±0.53 hours; control: 0.60±0.18; p=0.017) and the absolute deviation between actigraphy-derived bedtime from habitual bedtime (MDD: 0.95±0.78 hour, control: 0.45±0.15 hour; p=0.013). Accordingly, both indices were significantly larger in the MDD group as compared with the control group. Moreover, none of these sleep/circadian actigraphy indices was significantly associated with fractal activity correlation (all p values>0.05; Pearson or Spearman correlation test) or affected the observed group differences in fractal activity correlation during the last 3 hours of sleep preceding wake-up.

**Table 1:** Rest-activity patterns in MDD and control group

Variable	MDD (N=14)	Control (N=9)	P value
	Mean±SD	Mean±SD	
Sleep/wake timing and regularity			
SRI (%)	93.30±2.95	95.02±1.54	0.117
Habitual wake-up time (HH:MM)	8:00±0:58	7:34±1:19	0.364
Real wake-up time (HH:MM)	7:59±0:43	7:40±0:22	0.118
Real bedtime (HH:MM)	0:23±0:41	23:45±1:07	0.520
Absolute deviation of actual wake-up time from habitual wake time (hr)	0.95±0.53	0.6±0.18	0.017*
Absolute deviation of actual bedtime from habitual bedtime (hr)	0.95±0.78	0.45±0.15	0.013*
Average sleep duration (hr)	7.65±0.53	7.85±0.51	0.310
Daily activity rhythm			
Mean activity level	479.29±135.39	306.27±165.63	0.009*
24 hours amplitude	371.00±100.19	226.00±129.38	0.006*
24 hours amplitude†	0.78±0.10	0.82±0.12	0.466
24 hours acrophase (HH:MM)	15:39±0:43	15:30±1:10	0.817
Inter-daily stability	0.43±0.08	0.49±0.08	0.064
Intra-daily variability	0.80±0.15	0.80±0.14	0.826
M10 level†	1.03±0.12	1.06±0.19	0.546
M10 mid time (HH:MM)	15:03±0:51	15:37±2:13	0.446
L5 level†	0.02±0.01	0.02±0.01	0.685
L5 mid time (HH:MM)	3:56±0:45	3:39±0:50	0.475
Sleep duration/quality			
Efficiency (%)	85.82±4.69	86.93±4.43	0.600
Wake time during sleep (min)	54.29±19.13	42.37±24.82	0.260
Proportion of wake (%)	11.81±4.14	9.00±5.07	0.200
Proportion of wake (%) during the 0–3 hours before habitual wakeup	0.10±0.06	0.13±0.05	0.296

P values were from the statistical test for the group differences in the variable.

* Indicates statistically significant at alpha=0.05.

† Parameter normalised by the SD of all activity for each recording.

L5, an individual’s least active 5 hour; M10, an individual’s most active 10 hours; MDD, major depressive disorder; SRI, sleep-wake regularity index.

### Correlation between pre-wakeup fractal activity correlation, depressive symptom severity and melatonin levels

**Figure 2:**
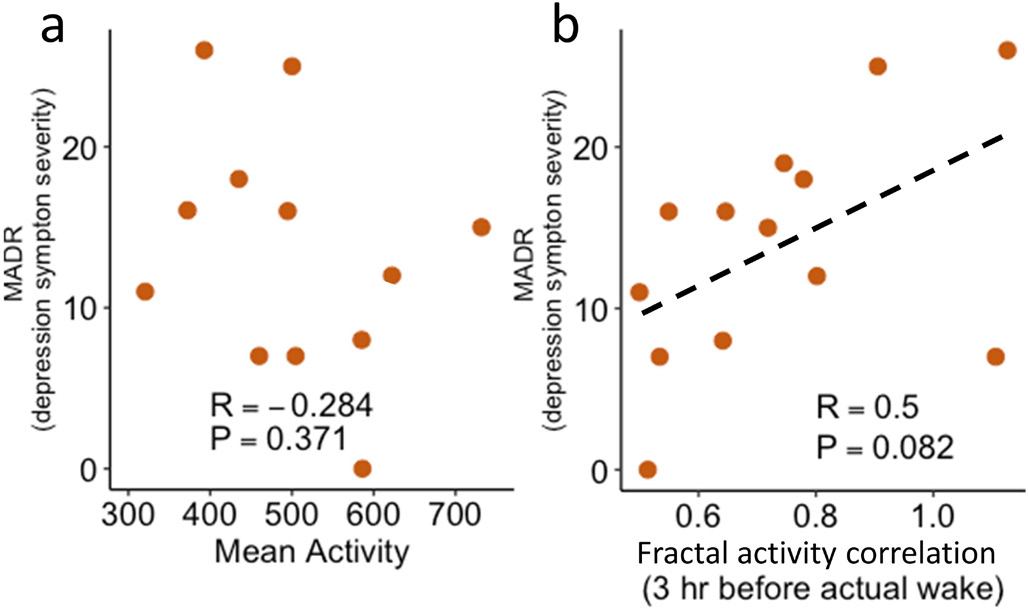
Associations of MADR with mean activity level (**a**) and fractal activity correlation during the last 3 hours before actual wake-up (**b**). The dashed line in (**b**) was obtained from the linear regression. The fitted line in (**a**) was not presented because the regression was not significant. MADR, Montgomery-Åsberg Depression.

Within the MDD group, we found that depressive symptom severity (MADR scale) tended to be positively associated with fractal activity correlation in the last 3 hours of actual sleep (R^2^=0.25, slope=17.74±20.37, p=0.08, figure 2b) but not mean activity level (p>0.05, figure 2a).

### Melatonin measures

**Figure 3:**
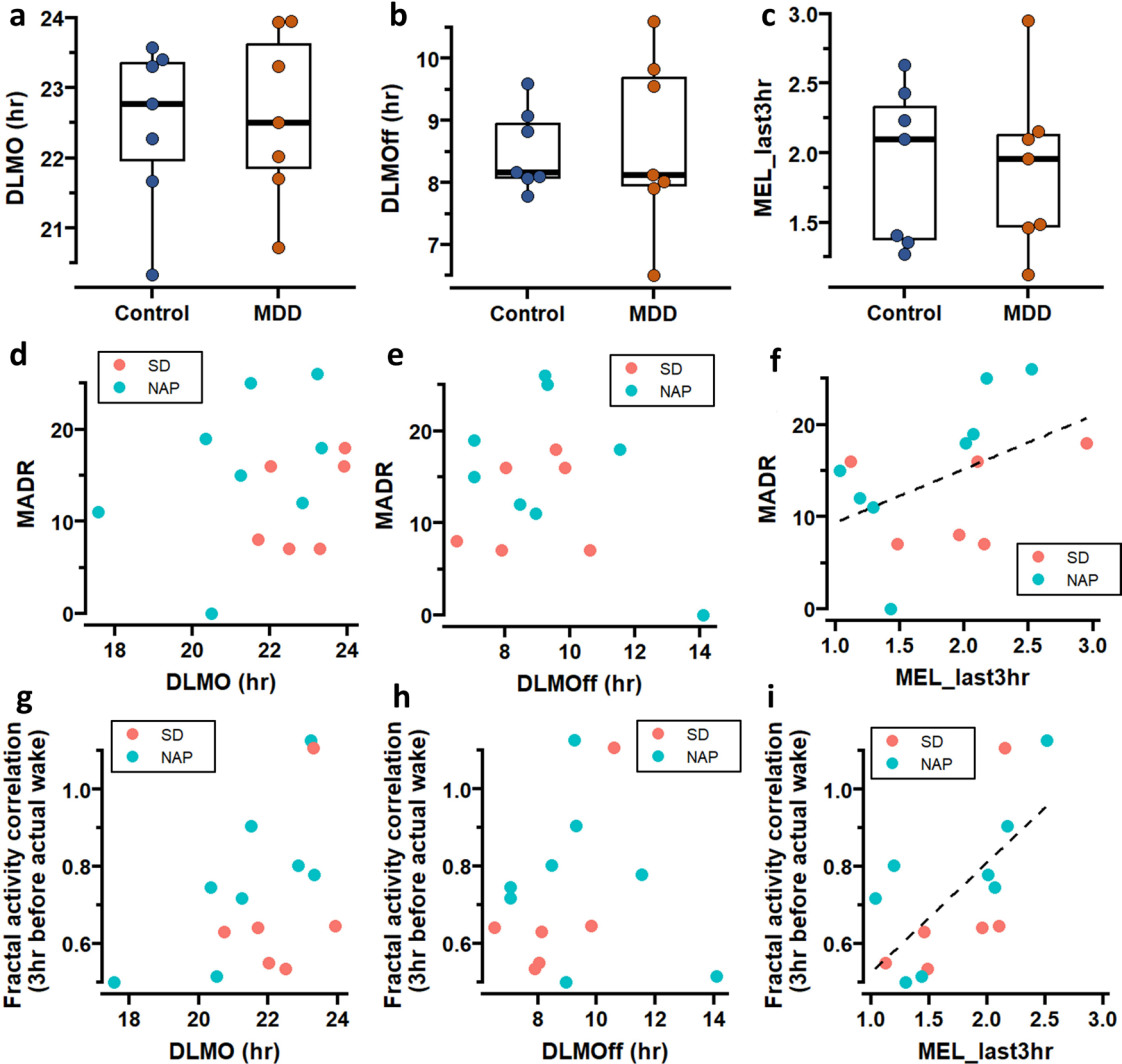
Melatonin analysis. (**a–c**) Group differences in DLMO (**a**), DLMOff (**b**) and MEL_last3hr (**c**). (**d–f**) Correlation between MADR and DLMO (**d**), DLMOff (**e**) and MEL_last3hr (**f**). (**g–i**) Correlation between fractal activity correlation during the last 3 hour before actual wake-up and DLMO (**g**), DLMOff (**h**) and MEL_last3hr (**i**). The dashed lines in (**f,i**) were obtained from the linear regression. The fitted line in (**d,e,g,h**) was not presented because the regression was not significant. DLMO, dim-light melatonin onset; MADR, Montgomery-Åsberg Depression.

We compared group difference of participants who underwent the circadian protocol with 40 hours sleep deprivation (SD protocol) (online supplemental table S1). The DLMO in the MDD and control group was 22:36±1:12 (HH:MM) and 22:30±1:10, respectively; DLMOff in the MDD and control group was 8:3±1:25 and 8:31±0:40, respectively; MEL_last3hr was 1.89±0.60 and 1.92±0.56, respectively. No group differences were found in DLMO, DLMOff or MEL-last3hr (all p>0.05, figure 3a–c). The phase angle between DLMO and bedtime was 1:47±0:23(SE) in the MDD and 1:36±0:21 in the controls; the phase angle between DLMOff and wake-up time was −0:23±0:33 (SE) in the MDD and −0:37±0:36 in the controls group, respectively. No group differences were found in these two phase angles (p=0.67 and 0.77, respectively, for the DLMO and bedtime and DLMOff and wake-up time, online supplemental table S1).

Within the MDD group, no significant correlations were found between the MADR and DLMO or DLMOff (adjusted R^2^=0.02 and 0.07, p=0.25 and 0.46, respectively, figure 3d,e), while a trending association between MADR and MEL_last3hr was found (adjusted R^2^=0.31, p=0.07). There were no significant correlations between DLMO or DLMOff and fractal activity correlation during the last 3 hours of habitual sleep (adjusted R^2^=0.22 and 0.07, p=0.56 and 0.74, respectively, figure 3g,h). There was a trend towards a positive correlation between the MELlast_3 hours and fractal activity correlation: for each unit increase in the MEL_last3hr (ie, higher relative melatonin level in the last 3 hours of sleep as compared to the 24 hours), the fractal activity correlation increased by 0.33±0.17 (SE) (p=0.08, figure 3i). On the other hand, the correlation between mean activity level and the melatonin levels was not significant (adjusted R^2^=−0.16, –0.15, and 0.01, respectively, for DLMO, DLMOff and MEL_last3hr, all p>0.10).

## Discussion

In this study, we found a higher fractal activity correlation in the women with MDD during the prewake windows, indicating a more wake-like pattern for these subjects. We also observed a trend for the fractal activity correlation to be positively correlated with depressive symptoms. While certain other actigraphy measures included mean activity level and absolute deviation of actual wake/bed time from habitual wake/bed time also showed a group difference between MDD and controls, these indexes did not show further correlation with the depressive symptoms. Furthermore, we also found a trend that the prewake fractal activity correlation in the women with MDD was positively correlated with the prewake melatonin level.

Disturbances in sleep and wakefulness are bidirectionally intertwined with major depression.^24^ While progress has been made in identifying associations between sleep, wakefulness and depression, our understanding of potential mechanisms underlying these links remains rudimentary. Our findings suggest that traditional linear measures of rest-activity patterns and sleep quality may not fully capture the discrete changes occurring in individuals at the onset of MDD, emphasising the benefit of using non-linear approaches as fractal measures. Our results indicate that unique changes in dynamic fractal activity patterns in women at the onset of MDD occurred specifically during sleep, especially the last 3 hours of sleep—a window akin to early morning awakenings in severe depression.^25^ Moreover, we showed a potential link between preawakening fractal activity correlation and depressive symptom severity. The observed increased mean activity level during wakefulness in our MDD patients was unexpected because reduced activity levels were usually reported in patients with MDD.^7 8^ Future studies are warranted to further clarify how different depression subtypes, disease duration and antidepressant treatment affect motor activity in MDD patients.

We showed for the first time a daily 24-hour rhythm of fractal activity correlation. Though reduced activity correlations or more random activity fluctuations during sleep might be expected (due to suppressed motor function in normal sleep), the finding of stronger fractal activity correlation before wake-up in the women with MDD is intriguing, suggesting more ‘wake-like’ temporal activity patterns at the end of sleep episodes. One intuitive explanation is the higher prevalence of sleep disturbances in MDD, including increased nocturnal awakenings, shortened REM (rapid eye movement) sleep onset latency and early morning awakening (terminal insomnia). However, this study excluded participants with sleep disorders confirmed by structured clinical interview, the PSQI and a PSG screening during the adaptation night. In addition, we excluded participants with other co-occurring psychiatric illnesses such as anxiety that are known to contribute to emotional dysregulation and fragmented REM sleep.^26^ These exclusions reduced the likelihood that the ‘wake-like’ sleep before habitual wake-up time was due to insomnia. Moreover, no group differences in the mean activity levels during the night (figure 1c) and in wake duration across the entire sleep episode or in the last few hours before habitual waking further argue against the insomnia explanation. Still, it is possible that the ‘wake-like’ fractal activity patterns were contributed by certain subtle or early-stage (undiagnosed) insomnia in our patients with MDD that might affect sleep occasionally (only certain days during the actigraphy assessment but not the PSG screening night) and could not be detected with actigraphy-based sleep scoring. Longer monitoring of these patients with MDD, ideally with simultaneous PSG and actigraphy assessments, is required to formally clarify this insomnia explanation.

An alternative mechanism underlying the altered fractal activity patterns before waking may be related to changes in the activation of motor activity control during the transition from sleep to wake-up and/or a state of hyperarousal during sleep, postulated to occur in depression.^25^ Such an alteration might not be detected by traditional actigraphy measures, supporting the idea that fractal activity measures might be more sensitive to certain intrinsic neuropathological changes.^21^ This is further supported by our findings that depression severity tended to be associated with the altered fractal activity correlation during sleep within the MDD group but not with any other traditional sleep/circadian measures, including irregular sleep/wake timing.

To assess the daily rhythm of temporal activity correlation, we examined activity data in eight 3-hour bins across the 24-hour sleep–wake cycle. In general, smaller sized bins or higher temporal resolution should better capture transient changes over time, especially when the changes are prior unknown. The selection of 3 hours was a compromise for reliable estimation of temporal correlation with DFA given the 2 min sampling. To precisely determine when fractal activity fluctuations start to show the group differences before wake-up, motor activity recordings with a higher sampling rate and/or improved analytical tools that can reliably assess temporal correlations from short recordings with less data points are required.

Circadian misalignment and sleep disturbances are thought to play a central role in the pathogenesis of depression.^27^ Melatonin secretion patterns related to sleep–wake phases appear to be different in patients with depression. Mellony *et al*^28^ found shorter phase angles between DLMO and midsleep in patients with bipolar disorder, indicating an earlier sleep phase relative to the onset of melatonin secretion. In patients with MDD, a shorter phase angle was also associated with more severe depression symptoms.^29^ In our study, the comparison of circadian phase using DLMO and DLMOff from salivary melatonin concentrations showed no statistically significant differences between MDD and healthy controls, as was previously also shown in a subset of the same women (using the SD protocol).^16^ Similarly, the phase angles between DLMO and bedtime, and DLMOff and wake-up time were not significantly different between the two groups. This might be due to the fact that women with MDD in our sample were enrolled around their first MDD episode when sleep disturbances and/or shortening of phase angles may not yet be manifested. In addition, all study participants were required to maintain a regular sleep–wake cycle for at least 7 days prior to entering the laboratory, which may also mask potential phase angle differences.

While our study provides valuable insights by focusing on unmedicated patients at the onset of MDD and utilising a rigorous circadian protocol to measure endogenous melatonin, several limitations must be acknowledged. First, the sample size was limited and only women were recruited, which may restrict the generalisability of our findings. Additionally, the age range of participants was narrow, which could impact the applicability of our results to a broader population. The types of sleep disturbance shown in different age groups might be different. A previous study showed that younger patients (18–35 years) with MDD commonly experience insomnia, while older patients (35–65 years) tend to have hypersomnia.^30^ Our findings of more ‘wake-like’ pattern detected by wearables may not be applied to a different age group. Future studies with larger sample sizes including a more diverse representation of the general population (ie, different genders, age and ethnoracial background) will help establish the role of actigraphy-based analytical tools including fractal analyses in detecting depression and assessing the depression-related behavioural changes. In addition, there are other non-linear analytical tools that can be used to assess temporal correlations and other complex patterns such as entropy and similarity in motor activity fluctuation^10 11^. Future studies should test and compare the performance of these different non-linear measures/tools in detecting MDD and other mood disorders, especially at the early phase or onset of the diseases.

Wearable technologies provides scalable, low-cost, low-burden yet powerful tools for determining sleep–wake patterns in the real world settings, representing a potential new avenue in remote medicine and the study of daily behaviours in patients with depression. Our findings emphasise the sensitivity of temporal fractal correlations to daily activity changes in patients at the onset of MDD. Thus, the fractal biomarker of daily activity holds a promising, cost-effective approach for detection and long-term monitoring of patients with early onset depression, including adolescent populations, in whom early detection and intervention promise the most significant benefit.

### Clinical Implications

Our study demonstrates that dynamic motor activity patterns can detect unique changes at the onset of MDD. We show that wearable technologies offer potential for early recognition and prevention of depression in young women. Specifically, fractal measures from actigraphy data may serve as a cost-effective biomarker for early identification and long-term monitoring of MDD.

## Supplementary material

10.1136/bmjment-2024-301321 online supplemental file 1

## Data Availability

Data are available upon reasonable request.
