# Supplementary material for: Fractal biomarker of daily activity for women with early onset depression
Source: BMJ Ment Health. 2025 Jun 10;28(1):e301321. doi: 10.1136/bmjment-2024-301321 (PMC12161324; doi:10.1136/bmjment-2024-301321)
Supplement: online supplemental file 1 [file bmjment-28-1-s001.pdf]

Supporting Material for

Fractal biomarker of daily activity for women with early onset depression

Hui-Wen Yang, Mirjam Münch, Ma Cherrysse Ulsa, Arlen Gaba, Angelina Birchler-Pedross, Sylvia Frey, Vera Knoblauch, Peng Li, Sarah Laxhmi Chellappa*, Christian Cajochen*, Kun Hu*

*Corresponding Author to: Kun Hu, Christian Cajochen, and Sarah Laxhmi Chellappa
Email: khu1@bwh.harvard.edu (K.H.); Christian.Cajochen@upk.ch (C.C.);
sarah.chellappa@outlook.com (S.L.C.).

This PDF file includes:

Supplementary Figure S1, S2, S3
Supplementary Table S1
Supplementary Material and methods
SI References

Supplementary Figure

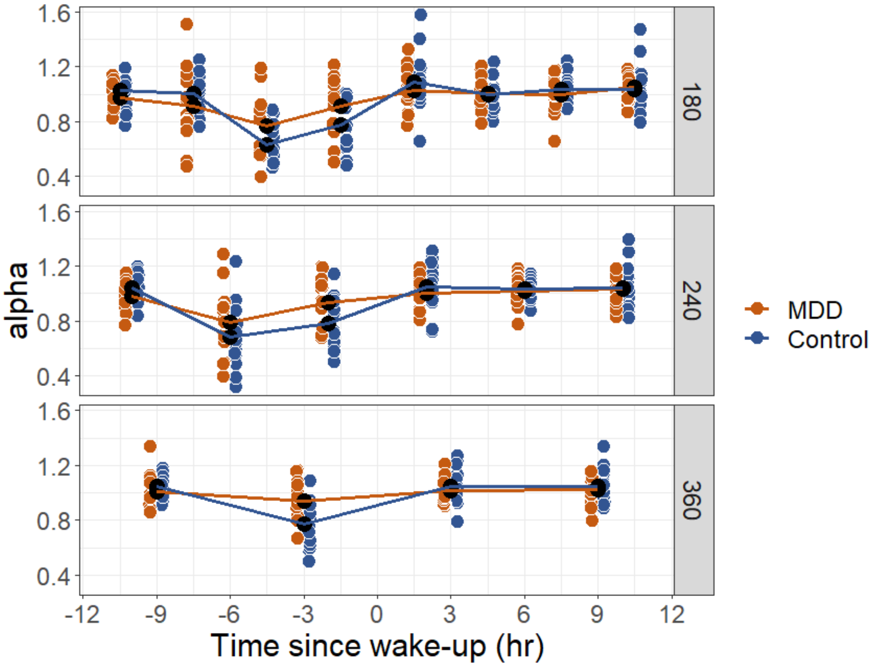

Figure S1. Daily rhythm of alpha. Results were obtained using different bin sizes across the 24-h sleep-wake cycle: (Top Panel) 3-h, (Middle Panel) 4-h bins, and (Bottom Panel) 6-h bins. Data were aligned relative to habitual wake-up time.

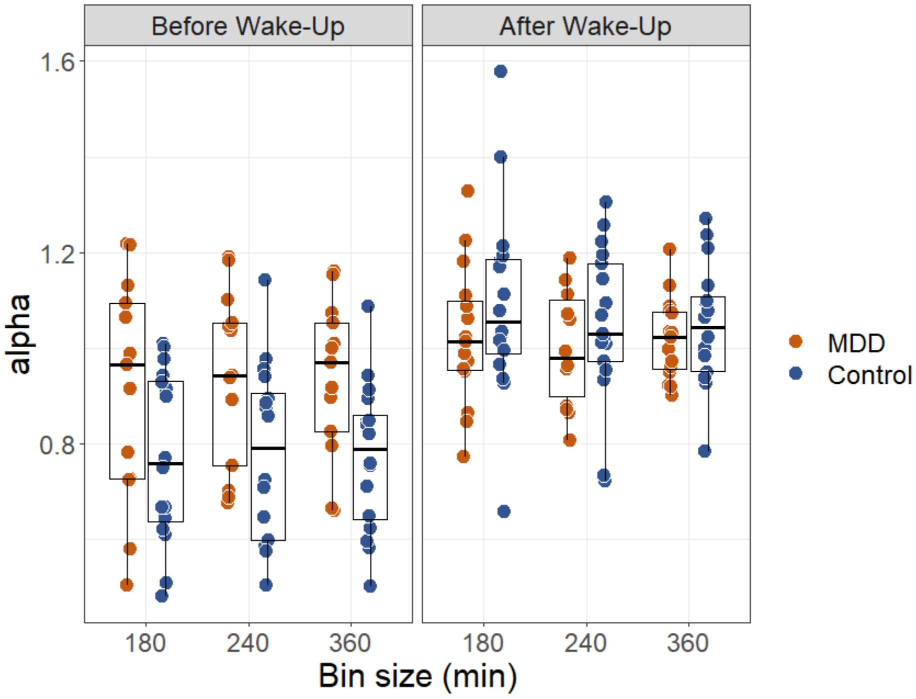

Figure S2. Group differences in alpha before and after habitual wake-up. Shown are the results in the last bins before wake-up (Left Panel) and the first bins after wake-up (Right Panel) from Figure S1.

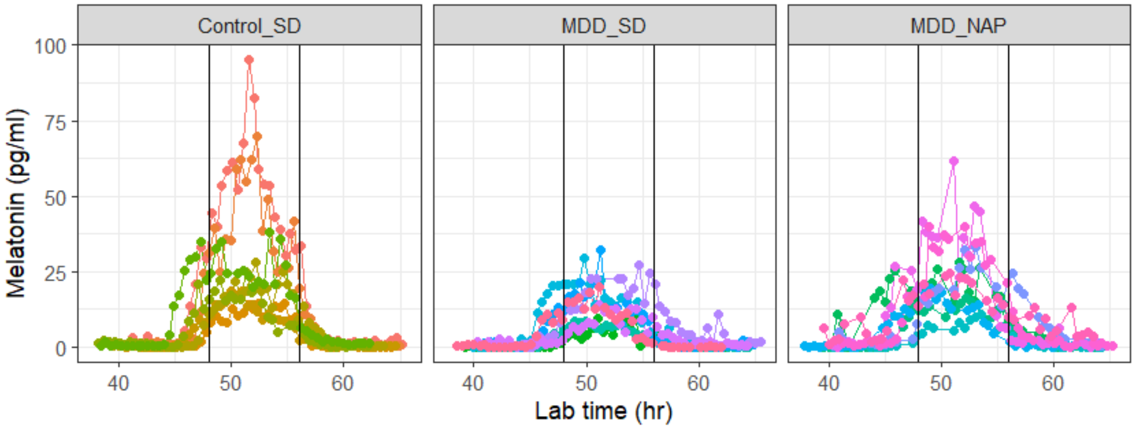

Figure S3. Melatonin secretion in the lab protocol. The data is aligned by the lab protocol, where sleep is scheduled for 8 hours by the subject’s habitual sleep and wake-up time (vertical lines).

Supplementary Table

Table S1: Melatonin measures and phase angles in MDD and control group during the SD protocol.

	MDD (N=7)			Control (N=8)			P-value
Variable	Mean		SD	Mean		SD	
DLMO (HH:MM)	22.36	±	1:12	22:30	±	1:10	0.89
DLMOff (HH:MM)	08:39	±	1:25	08:31	±	0:40	0.86
MEL_last3hr	1.89	±	0.60	1.92	±	0.56	0.94
Phase angle	Mean		SE	Mean		SE	P-value
DLMO and bed time (HH:MM)	1:47	±	0:23	1:36	±	0:21	0.67
DLMOff and wake-up time (HH:MM)	-0:23	±	0:33	-0:37	±	0:36	0.77

Supplementary Materials and Methods

Participants

Different aspects of this study designed to test separate hypotheses have been published previously.[1–5] All procedures conformed to the Declaration of Helsinki and the local Ethical Committee (Ethikkommission beider Basel, policy number: 13.895.522; study number: 07.064). Due to the unbalanced prevalence of MDD in different genders (women are twice as likely to experience MDD as men)[6,7] and the difficulty in recruiting participants at the early onset of the disease without co-morbid sleep disturbances, only women were included in this study. All these young women with MDD underwent a clinical interview with the same clinical psychologist and met the following criteria:

1. They experienced an episode of MDD when undertaking the study and fulfilled the diagnostic criteria of the Diagnostic and Statistical Manual (DSM-IV) for MDD.[8]
2. They had neither atypical symptoms nor other co-morbid psychiatric DSM IV disorders.
3. They had no severe sleep problems as measured by the Pittsburgh Sleep Quality Index (PSQI < 8)[9] and were not evening types as measured with the morning-evening-type questionnaire.[10]
4. They did not have a long history of depression and had the current episode <24 months before the study date.
5. They did not receive psychiatric treatment and did not use psychotropic drugs.

During the clinical interview by the clinical psychologist, the structured assessment depression scales, including Hamilton-17[11] and the Montgomery-Åsberg Depression Scale[12] (MADRS; mean:16.71 ± 2.13 SD), and the Beck Depression Inventory[13] (BDI; mean value 21.29 ± 6.84 SD) were assessed.

In the original experiment design, 16 MDD participants and 9 age-matched control women were recruited. However, activity recordings were collected only from 15 MDD participants. In addition, one MDD participant who took the wristwatch off each night was removed from all the analyses related to daily rhythm and sleep. As a result, our case-control study included 14 young unmedicated women at MDD onset (age: 19-33 years; 24.9±5.2y [Mean ± SD] years) and nine age-matched healthy control women (age: 20-31 years; 24.8±3.5y).

Activity recording:

At home, motor activity levels were accessed for ~7 consecutive days using wrist-worn actigraphy (Cambridge Neurotechnology®, Cambridge, UK). The original data collection settings were inconsistent across patients, with some recorded in 1-minute epochs and others in 2-minute epochs. To ensure data harmonization and avoid potential bias due to different epoch length, we opted to resample all data to 2-minute intervals. All participants were instructed to maintain a regular sleep-wake cycle targeting 8 hours of sleep at self-selected times (habitual bed/wake). For the control group, 7 of the 9 participants had two recordings separated by 4 weeks. The rest-activity patterns and fractal analysis were performed separately for each recording.

Fractal analysis

We performed the detrended fractal analysis (DFA) to assess the temporal correlations in activity fluctuations at time scales between 20-40 minutes. A parameter, alpha, is derived from DFA with alpha = 0.5 indicating no correlation, i.e., white noise; larger alpha values indicating stronger temporal correlations. alpha ~1 is often observed in physiological signals of healthy systems[14–16] and is believed to represent the most complex fluctuation pattern with a delicate balance between being total random and being too rigid.[17,18] Details of the DFA and interpretation of alpha have been provided in previous studies[19–21]. Here, to investigate the daily rhythm of fractal correlations, we calculated alpha in each 3-hour bin across the sleep-wake cycle by either habitual wake-up time or actual wake-up time. For example, for a recording of a participant with habitual wake-up at 7 AM, we cut the time series into eight 3-h bins: 7AM-9:59AM, 10AM-12:59PM, 1PM-3:59PM, 4PM-6:59PM, 7PM-9:59PM, 10PM-0:59AM, 1AM-3:59AM, and 4AM-6:59AM. For each bin, the 3-hour segments from different days were combined and were analyzed using the DFA to

obtain alpha at time scales between 20-40 min. When using actual wake-up time, we considered only 9 hours before wake-up and 9 hours after wake-up (totaling six 3-h bins) because the sleep-wake cycle might not be exactly 24 h. For each 3-h bin, the 3-hour segments from different days were also combined to obtain one alpha at time scales between 20-40 min.

Rest-activity measures and sleep quality indexes

In addition to dynamic fractal activity patterns, we explored other sleep/circadian related rest-activity measures derived from actigraphy and habitual sleep/wake timing.

a. Mean activity level and cosinor analysis

The mean activity level (arbitrary units) was calculated using all the data points for each recording. In addition, we fitted each recording using a cosinor function of a 24-hr period and a 12-hr harmonic, and the 24-h component was used to determine the amplitude and acrophase (peak time) of the 24-hr rhythm. To eliminate the effect of different actiwatch devices, we normalized the amplitude of the 24-hr rhythm by the standard deviation of activity level for each recording.

b. Non-parametric measures of the 24-h activity rhythm

The interdaily stability (IS) and intradaily variability (IV)[22,23] are two non-parametric measures that quantify the stability and fragmentation of 24-hr activity rhythms, respectively. The IS value ranges from 0 to 1, with a higher value indicating stable or regular rest-activity patterns. The IV ranges between 0 and 2, and larger IV values indicate more fragmented rest-activity patterns. In this study, data were first resampled to epochs of 30 minutes (the time bin size) for the calculation of IS and IV. For each recording, the most active 10 hours (M10) and the least active 5 hours (L5) across the 24-h cycle were determined to obtain four measures:[22] M10 level (mean activity level during the 10 hours), L5 level (mean activity level during 5 hours), and the mid times of M10 and L5, respectively.

c. Sleep/wake timing and regularity

To understand the daily sleep/wake behaviors, we also determined the actual bedtime and wake-up time per night by integrating the opinions of 5 independent scorers who checked and corrected the results from the automatic detection algorithm provided by Actiware 5.52.0003 (Phillips Respironics, Bend, Oregon, USA). After excluding the sleep episodes from our analysis for which the 5 scorers could not reach a conclusion, or the subject took the wristwatch off before bedtime, we obtained the following sleep/wake timing and regularity indexes for each recording: (i) The Sleep Regularity Index (SRI) quantifies the regularity of the sleep-wake cycle.[24] The SRI value ranges from 0 (non-overlapped sleep and wake episodes between two adjacent cycles) to 100 (perfectly regular sleep-wake cycles). (ii) To further distinguish the separate contributions of bedtime and wake-up time to SRI, we calculated the standard deviations of bedtime and wake-up time across different days. (iii) For each of sleep episodes, we also calculated the absolute difference between actual bedtime and habitual bedtime, and the absolute difference between actual wake-up time and habitual wake-up time.

d. Sleep quality indexes.

After determining the onset and offset of each ‘actual’ sleep episode from actigraphy, we obtained the following sleep quality indexes from Actiware for each night: (i) Sleep duration, defined by the time lag between offset and onset of the whole-night sleep episode; (ii) total duration of wake (in minutes) during the sleep episode,; (iii) percentage of wake during the sleep episode (0-100%); and (iv) percentage of wake during the last 3 hours of habitual sleep (0-100%).

Selection of DFA parameters

In this study, we selected the time scale 20-40 min for the 3-hour bin. This bin size was selected to capture 24-hour rhythm in fractal activity patterns (i.e., the rhythm would be less or not visible if the size was too large) while providing sufficient data points in each bin for reliable estimation of temporal correlations in the fluctuations. We repeated the calculation of alpha values before wake-up using a different bin size (=4h and 6h). The similar alpha values and the group differences were obtained while the rhythm of alpha was attenuated (Supplementary Figure S1 and S2).

Regarding potential biases and inaccuracies in estimated correlations when performing DFA on a short recording, three main factors should be considered:

(1) Trends. Certain strong local trends may cause artificially increases in the DFA derived fluctuation amplitude at large time scales.[25] This occurs especially when the first order DFA is used. Note, to calculate the fluctuation amplitude F(n) at a time scale of n, a polynomial function is used to fit the integrated signal in each window of the same size of n; and the order of polynomial functions in this detrending step is the DFA order. Thus, it is generally recommended that the second order DFA or DFA-2 (i.e., 2nd order polynomial fitting) should be used when certain linear trends may be present.[25]

(2) Overfitting. For very small timescales, the detrending step in the DFA causes overfitting or more under-estimated variances or fluctuation amplitudes F(n) because the number of data points is closer to the number of parameters in the polynomial fitting. Thus, including F(n) at very small timescales might lead to overestimated correlations, especially for anticorrelated signals, as pointed out initially in the study of Hu et al.[25] and later studies.[26,27] To minimize the bias, it should be sufficient to exclude those F(n) at very small timescales when calculating the scaling exponent.[25] In this study, we excluded F(n) with n<10 points (or 20 min) which should be more than enough for reliable estimation of positive correlations in motor activity fluctuations (alpha >0.5). Other studies seemed not in favor of or ignored this simple solution. As an alternative approach, there were certain attempts to address the overestimation problem by modifying the calculation of F(n) in the DFA. For instance, ‘unbiased DFA’ was introduce by Yuan et al.[27] Though it was reported that the new approach provides more accurate estimation of temporal correlations, it is evident that the revised F(n) significantly deviates from a power-law function— the key mathematical manifestation of fractal or scaling behavior.

(3) Underpowered statistics. For a large timescale n (closer to the signal length), the estimated fluctuation amplitude F(n) will become unreliable (or statistically underpowered) because there are too few windows with the size of n to estimate mean fluctuation, which is similar to the case of obtaining a mean value from few data points. According to the simulation results,[25] the upper timescale n_{max} to obtain a reliable DFA scaling exponent should allow at least 10 non-overlapped windows with the size of n_{max}. Ignoring this will lead to huge variations in the DFA derived scaling exponents. In this study, we used n_{max} = 20 points (or 40 min) that allowed > 20 windows to calculate F(n) even after considering certain missing data.

SI References

1 Frey S, Birchler-Pedross A, Hofstetter M, *et al.* Challenging the sleep homeostat: sleep in depression is not premature aging. *Sleep Med*. 2012;13:933–45. doi: 10.1016/j.sleep.2012.03.008

2 Birchler-Pedross A, Frey S, Chellappa SL, *et al.* Higher frontal EEG synchronization in young women with major depression: a marker for increased homeostatic sleep pressure? *Sleep*. 2011;34:1699–706. doi: 10.5665/sleep.1440

3 Birchler-Pedross A, Frey S, Götz T, *et al.* Subjective Mood in Young Unmedicated Depressed Women under High and Low Sleep Pressure Conditions. *Biology*. 2016;5:E52. doi: 10.3390/biology5040052

4 Frey S, Birchler-Pedross A, Hofstetter M, *et al.* Young women with major depression live on higher homeostatic sleep pressure than healthy controls. *Chronobiol Int*. 2012;29:278–94. doi: 10.3109/07420528.2012.656163

5 Birchler-Pedross A, Frey S, Cajochen C, *et al.* Circadian and Sleep Modulation of Dreaming in Women with Major Depression. *Clocks & Sleep*. 2022;4:114–28. doi: 10.3390/clockssleep4010012

6 Schuch JJJ, Roest AM, Nolen WA, *et al.* Gender differences in major depressive disorder: results from the Netherlands study of depression and anxiety. *J Affect Disord*. 2014;156:156–63. doi: 10.1016/j.jad.2013.12.011

7 Gutiérrez-Rojas L, Porras-Segovia A, Dunne H, *et al.* Prevalence and correlates of major depressive disorder: a systematic review. *Braz J Psychiatry*. 2020;42:657–72. doi: 10.1590/1516-4446-2020-0650

8 Guze SB. Diagnostic and Statistical Manual of Mental Disorders, 4th ed. (DSM-IV). *AJP*. 1995;152:1228–1228. doi: 10.1176/ajp.152.8.1228

9 Buysse DJ, Reynolds CF, Monk TH, *et al.* The Pittsburgh sleep quality index: A new instrument for psychiatric practice and research. *Psychiatry Res*. 1989;28:193–213. doi: 10.1016/0165-1781(89)90047-4

10 Torsvall L, Åkerstedt T. A diurnal type scale: Construction, consistency and validation in shift work. *Scand J Work Environ Health*. 1980;6:283–90.

11 Williams JBW, Terman M. Structured Interview Guide for the Hamilton Depression Rating Scale with Atypical Depression Supplement (SIGH-ADS) New York State Psychiatric Institute. *New York*. 2003.

12 Montgomery SA, Åsberg M. A New Depression Scale Designed to be Sensitive to Change. *Br J Psychiatry*. 1979;134:382–9. doi: 10.1192/bjp.134.4.382

13 Beck AT, Ward CH, Mendelson M, *et al.* An inventory for measuring depression. *Arch Gen Psychiatry*. 1961;4:561–71. doi: 10.1001/archpsyc.1961.01710120031004

14 Bassingthwaighte JB, Liebovitch LS, West BJ. *Fractal Physiology*. American Physiological Society 1994.

15 Goldberger AL, Amaral LAN, Hausdorff JM, *et al.* Fractal dynamics in physiology: alterations with disease and aging. *Proc Natl Acad Sci U S A*. 2002;99 Suppl 1:2466–72. doi: 10.1073/pnas.012579499

16 West BJ. Fractal physiology and the fractional calculus: a perspective. *Front Physiol*. 2010;1:12. doi: 10.3389/fphys.2010.00012

17 Stanley HE, Buldyrev SV, Goldberger AL, *et al.* Fractal landscapes in biological systems: long-range correlations in DNA and interbeat heart intervals. *Physica A*. 1992;191:1–12.

18 Bak P, Tang C, Wiesenfeld K. Self-organized criticality: An explanation of the 1/f noise. *Phys Rev Lett*. 1987;59:381–4.

19 Peng CK, Havlin S, Hausdorff JM, *et al.* Fractal mechanisms and heart rate dynamics. Long-range correlations and their breakdown with disease. *J Electrocardiol*. 1995;28 Suppl:59–65.

20 Hu K, Ivanov PC, Chen Z, *et al.* Non-random fluctuations and multi-scale dynamics regulation of human activity. *Physica A*. 2004;337:307–18. doi: 10.1016/j.physa.2004.01.042

21 Xu LM, Ivanov PC, Hu K, *et al.* Quantifying signals with power-law correlations: A comparative study of detrended fluctuation analysis and detrended moving average techniques. *Phys Rev E*. 2005;71:051101. doi: 10.1103/PhysRevE.71.051101

22 Witting W, Kwa IH, Eikelenboom P, *et al.* Alterations in the circadian rest-activity rhythm in aging and Alzheimer's disease. *Biol Psychiatry*. 1990;27:563–72. doi: 10.1016/0006-3223(90)90523-5

23 Van Someren EJ, Kessler A, Mirmiran M, *et al.* Indirect bright light improves circadian rest-activity rhythm disturbances in demented patients. *Biol Psychiatry*. 1997;41:955–63. doi: 10.1016/S0006-3223(97)89928-3

24 Phillips AJK, Clerx WM, O'Brien CS, *et al.* Irregular sleep/wake patterns are associated with poorer academic performance and delayed circadian and sleep/wake timing. *Sci Rep*. 2017;7:3216. doi: 10.1038/s41598-017-03171-4

25 Hu K, Ivanov PC, Chen Z, *et al.* Effect of trends on detrended fluctuation analysis. *Phys Rev E*. 2001;64:011114. doi: 10.1103/PhysRevE.64.011114

26 Roume C, Ezzina S, Blain H, *et al.* Biases in the Simulation and Analysis of Fractal Processes. *Computational and Mathematical Methods in Medicine*. 2019;2019:4025305. doi: 10.1155/2019/4025305

27 Yuan Q, Gu C, Weng T, *et al.* Unbiased detrended fluctuation analysis: Long-range correlations in very short time series. *Physica A: Statistical Mechanics and its Applications*. 2018;505:179–89. doi: 10.1016/j.physa.2018.03.043
